# Sequencing analysis of SARS-CoV-2 cases in Slovenian long-term care facilities to support outbreak control

**DOI:** 10.3389/fpubh.2024.1406777

**Published:** 2024-05-15

**Authors:** Rok Kogoj, Manja Grašek, Alen Suljič, Samo Zakotnik, Doroteja Vlaj, Kaja Kotnik Koman, Mario Fafangel, Miroslav Petrovec, Tatjana Avšič-Županc, Misa Korva

**Affiliations:** ^1^Institute of Microbiology and Immunology, Faculty of Medicine, University of Ljubljana, Ljubljana, Slovenia; ^2^Communicable Diseases Center, National Institute of Public Health, Ljubljana, Slovenia

**Keywords:** SARS-CoV-2, next-generation sequencing, long-term care facility, outbreak control, phylogeny

## Abstract

**Introduction:**

Residents of long-term care facilities (LTCFs) are at high risk of morbidity and mortality due to COVID-19, especially when new variants of concern (VOC) emerge. To provide intradisciplinary data in order to tailor public health interventions during future epidemics, available epidemiologic and genomic data from Slovenian LTCFs during the initial phases of the COVID-19 pandemic was analyzed.

**Methods:**

The first part of the study included SARS-CoV-2 reverse-transcription Real-Time PCR (rtRT-PCR) positive LTCF residents, from 21 facilities with COVID-19 outbreaks occurring in October 2020. The second part of the study included SARS-CoV-2 rtRT-PCR positive LTCF residents and staff between January and April 2021, when VOC Alpha emerged in Slovenia. Next-generation sequencing (NGS) was used to acquire SARS-CoV-2 genomes, and lineage determination. In-depth phylogenetic and mutational profile analysis were performed and coupled with available field epidemiological data to assess the dynamics of SARS-CoV-2 introduction and transmission.

**Results:**

370/498 SARS-CoV-2 positive residents as well as 558/699 SARS-CoV-2 positive residents and 301/358 staff were successfully sequenced in the first and second part of the study, respectively. In October 2020, COVID-19 outbreaks in the 21 LTCFs were caused by intra-facility transmission as well as multiple independent SARS-CoV-2 introductions. The Alpha variant was confirmed in the first LTCF resident approximately 1.5 months after the first Alpha case was identified in Slovenia. The data also showed a slower replacement of existing variants by Alpha in residents compared to staff and the general population.

**Discussion:**

Multiple SARS CoV-2 introductions as well as intra-facility spreading impacted disease transmission in Slovenian LTCFs. Timely implementation of control measures aimed at limiting new introductions while controlling in-facility transmission are of paramount importance, especially as new VOCs emerge. Sequencing, in conjunction with epidemiological data, can facilitate the determination of the need for future improvements in control measures to protect LTCF residents from COVID-19 or other respiratory infections.

## 1 Introduction

Since the emergence of SARS-CoV-2, various types of long-term care facilities (LTCFs) have experienced the majority of the COVID-19 burden. In the United States alone, more than 2 million documented cases have resulted in more than 160,000 deaths among residents and nearly 3,000 deaths among staff in LTCFs (1, 2). A similar situation has been observed in other parts of the world (3), including South America (4), Canada (5), the European Union (6–9), England (10), China (11), and Australia (12). The combination of vulnerable populations, staff shortages due to sickness leave, insufficient resources for rapid and accurate testing, and lack of personal protective equipment has created a situation referred to as the “perfect storm” (13).

Non-pharmaceutical measures such as wearing masks, physical distancing, quarantine/isolation, frequent hand washing, wiping surfaces with disinfectants, not touching the eyes, nose, and mouth, and sneezing or coughing into elbows have been used to quell this storm and have played an important role in slowing the spread of SARS-CoV-2 in these settings (14). Non-pharmaceutical measures have been implemented in LTCFs around the world in various combinations and strategies depending on the pandemic wave. Most commonly, a combination of wearing personal protective equipment and regular screening of residents, staff, and visitors, regardless of their respiratory symptoms, has been used (15). An immense step forward in the fight against COVID-19 was the vaccines that were developed with unprecedented effort and became available toward the end of 2020 (16). In most EU/EEA countries, COVID-19 vaccination programs have prioritized LTCF residents and identified them as one of the main focus groups for immunization. With the gradual increase in vaccination rates among LTCF residents, there has been a significant reduction in morbidity and mortality in this population (17). However, during the COVID-19 pandemic, several variants of SARS-CoV-2 emerged, some of which were classified as variants of concern (VOC) due to greater transmissibility, ability to cause more severe disease and higher mortality, or enhanced ability to evade natural or vaccine-derived immunity (18). In already high-risk environments such as LTCFs, these variants pose an even greater threat to the wellbeing of the residents while also posing a greater challenge to effective containment.

Next,-generation sequencing (NGS) has proven to be a powerful tool to generate data in the investigation of the COVID-19 pandemic and to contribute to the scientific understanding and public health response (19). Alongside its numerous advantages, it can assist in the investigation of SARS-CoV-2 transmission dynamics in healthcare settings, which can reveal clusters of infection (20, 21). However, NGS alone might not be enough to understand the directionality of spread or low genomic diversity indicating possible spurious transmission events (22).

The aim of this study was to interpret genomic sequencing data obtained in LTCFs to determine virus introduction pathways and to support future mitigation measures, especially in the case of the emergence of viral variants. The first part of the study assessed whether one or multiple introductions of SARS-CoV-2 occurred within the respective LTCFs during October 2020 (the second pandemic wave). Furthermore, we performed a detailed complete genome analysis of SARS-CoV-2 cases in one LTCF at the onset of its first outbreak to increase the resolution of the results. In the second part of the study, we aimed to determine how early VOC Alpha emerged in LTCF residents and to describe the substitution of existing lineages with the more transmissible Alpha variant in these high-risk environments, where more stringent measures were in place compared to the general population.

## 2 Materials and methods

### 2.1 Study population and sample selection

In this study, residents and staff from Slovenian LTCFs were included. The vast majority (over 90% of included residents and staff) were from LTCFs that provide care for the older adult, and the minority (under 10% of included residents and staff) were from LTCFs that provide institutional care for adult individuals with physical or mental developmental disabilities, mental health issues, sensory impairments and mobility disorders. The study was divided into two parts.

In the first part, all LTCF residents that tested positive for SARS-CoV-2 in October 2020 and whose samples were analyzed by the Institute of Microbiology and Immunology (IMI) of the Faculty of Medicine, University of Ljubljana, were included. Residents also had to be part of an outbreak in a LTCF, defined as at least two confirmed COVID-19 cases within 14 days. Following these criteria, residents from 21 LTCFs were included. In addition, one specific LTCF was selected in which SARS-CoV-2–positive residents were detected in the period of 1 month without known prior SARS-CoV-2 infections and before vaccination was available.

**Figure 1:**
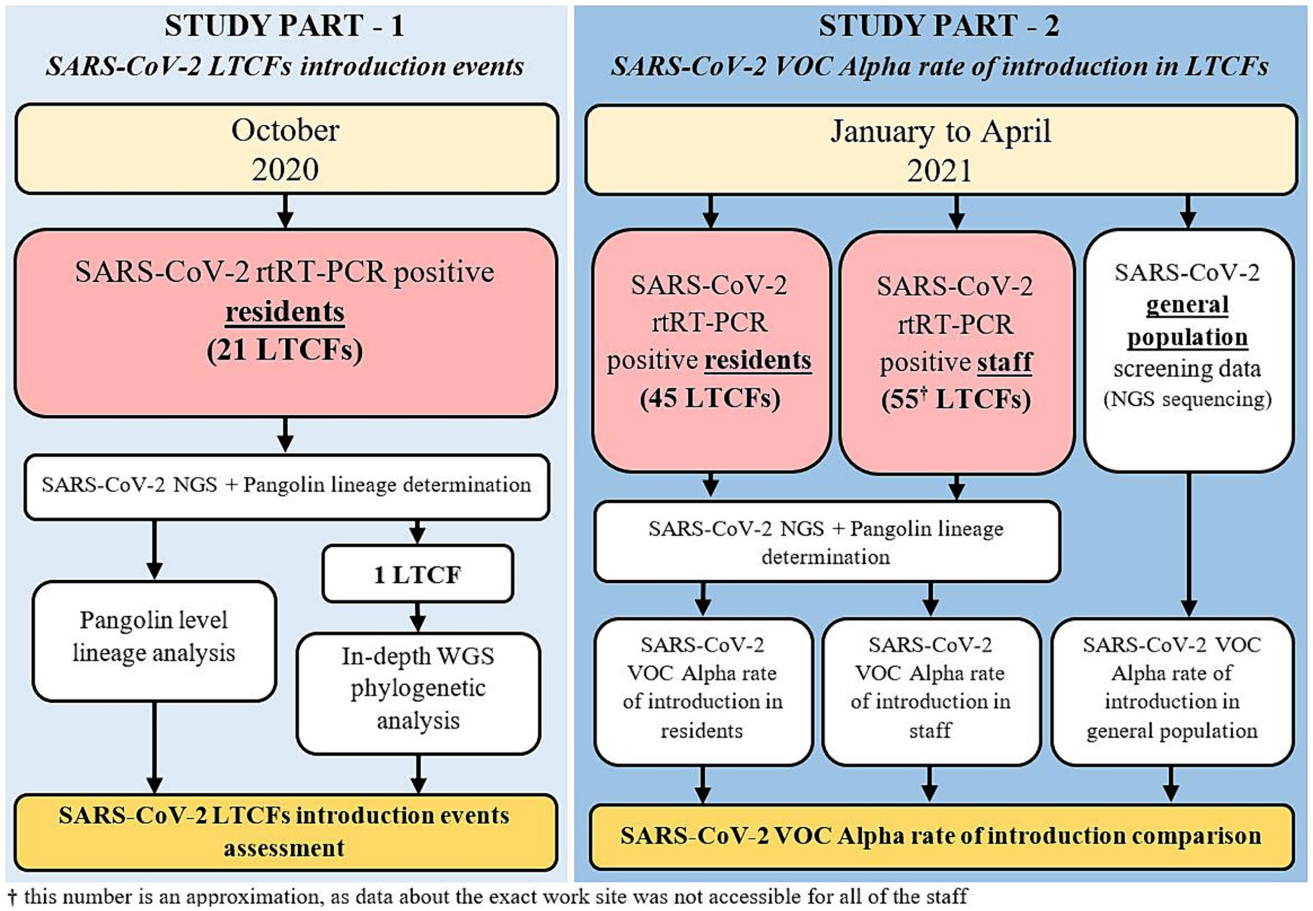
Study design flowchart.

In the second part of the study, all LTCF residents and staff that tested positive for SARS-CoV-2 from January to April 2021 and whose samples were analyzed by the IMI were included. This included residents from 45 LTCFs and staff from approximately 55 LTCFs (data about the exact work site was not accessible for all of the staff). LTCF staff were defined as anyone that was registered in the national digital administrative database as an employee in a LTCF. A detailed schematic of the study design is shown in Figure 1 and Table 1.

**Table 1:** Detailed study characteristics.

Study part	Number of included LTCFs	Time period	Patient category	PCR testing site	SARS-CoV-2 cases
1	21	October 2020	Residents	IMI	498
2	45	January to April 2021	Residents	IMI	699
	55^a^		Staff	IMI	358

a This number is an approximation, as data about the exact work site was not accessible for all of the staff.

Samples from the selected individuals were nasopharyngeal swabs collected onsite at respective LTCFs by healthcare professionals in Viral Transport Media VTM; (Liofilchem, Roseto degli Abruzzi, Italy) and immediately sent to the IMI via courier for SARS-CoV-2 detection by rtRT-PCR. Samples were tested the same day and then stored at −80°C until the time of the study.

### 2.2 Sample preparation, library creation, and next-generation sequencing

Total nucleic acids (NA) were isolated from 300 μL of nasopharyngeal swabs collected in VTM. Pure NA eluates were obtained using the OptiPure Viral Auto Plate, Proteinase K Reagent Kit (TANBead, Taoyuan City, Taiwan) on a Maelstrom 9,600 instrument (TANBead, Taoyuan City, Taiwan).

Isolated nucleic acids were prepared for NGS using COVIDSeq Test (Illumina, San Diego, CA, United States) following the manufacturer’s instructions. The concentration of each library was measured using the Qubit dsDNA HS Assay Kit (Thermo Scientific, Waltham, MA, United States), and fragment size was measured using the Agilent High Sensitivity DNA Kit (Agilent Technologies, Santa Clara, CA, United States). Finally, sequencing was performed using the MiSeq Reagent Kit v3 (600 cycles) on a MiSeq sequencer or the NextSeq 500/550 High Output Kit v2.5 (300 cycles) on a NextSeq 550 sequencer (Illumina, San Diego, CA, United States).

### 2.3 Data analysis

The in-house developed bioinformatics pipeline consisted of initial trimming of reads with BBDuk (v38.96) and quality control of raw data using FastQC (v0.11.5). The filtered reads were mapped to the reference genome of the Wuhan Hu-1 isolate (NCBI accession number NC_045512.2) using BWA-MEM (v0.7.17-r1188). Subsequent data processing, including sorting, partner tagging, duplicate tagging, indexing, and coverage depth calculation, was performed using Samtools (v1.9) (23). Variant calls were generated using iVAR (v1.3.1) (24) with the following settings: minimum quality score threshold for base counting set to 20, minimum frequency threshold set to 0.01, and minimum read depth for variant calling set to 10.

Lineages were assigned based on the consensus sequences of near-complete SARS-CoV-2 genomes using the Pangolin tool (v4.2), which implements a dynamic nomenclature (25). The consensus sequences were additionally aligned with MAFFT (v7.490) using a global alignment over 1,000 iterations (26). The construction of the phylogenetic tree based on the multiple sequence alignment was performed using IQ-TREE 2 (v2.2.0) with automatic model selection for tree inference and ultra-fast bootstrap with 1,000 iterations (27). Data analysis was performed with R statistical software version 4.3.0 (R Foundation for Statistical Computing, Vienna, Austria) and Microsoft Excel 2019 version 1801 (Microsoft, Redmond, WA, United States).

### 2.4 Ethical approval

This study was performed in accordance with the ethical guidelines for human research, the World Medical Association’s Declaration of Helsinki, the Oviedo Convention on Human Rights and Biomedicine, and the Slovenian Code on Medical Deontology. The study was approved by the National Medical Ethics Committee, Ministry of Health, Republic of Slovenia (0120 211/2020/7). The need for informed patient consent was waived, as the manuscript contains no identifying information or images.

## 3 Results

### 3.1 Overall distribution of SARS-CoV-2 lineages in LTCFs

Of the 498 COVID-19 positive LTCF residents in the first period of the study, 74.3% (370/498) were successfully sequenced. Of the 699 and 358 COVID-19 positive LTCFs residents and staff in the second period of the study, 79.8% (558/699) and 84.1% (301/358) were successfully sequenced, respectively. Samples for which complete SARS-CoV-2 genome was not obtained, originated from patents with low viral loads and were consequently excluded from further analyses.

In the first study period (October 2020), nine lineages were observed among the residents of the 21 selected LTCFs. The most common lineage was B.1.1.70 with 35.6%, followed by B.1.160 with 32.6% and B.1.258.17 with 18.2%. Six other lineages were present among the residents, which together accounted for the remaining 13.6%.

In the second study period (January to April 2021), 10 lineages were detected among residents and 17 among staff. Lineage B.1.258.17 was the most abundant lineage in both subpopulations, at 65.2 and 63.6% in residents and staff, respectively. Among staff, Alpha is already the second most common lineage at 16.9% in contrast to residents, where Alpha is only the fifth most common lineage (5.0%). Table 2 shows a detailed representation of sequencing results and the lineages detected in both time periods and subpopulations.

**Table 2:** Detailed overall sequencing results for both study periods.

Study period		1	2	
Time period		October 2020	January – April 2021	
No. of assessed LTCFs		21	45	55^a^
Sample provenience		Residents	Residents	Staff
No. PCR confirmed cases		498	699	358
% (No.) sequenced		74.3% (370)	79.8% (558)	84.1% (301)
SARS-CoV-2 variant	A.27	N/A	0.2% (1)	1.0% (3)
	B.1	3.5% (13)	0.2% (1)	0.7% (2)
	B.1.1	0.3% (1)	N/A	0.7% (2)
	B.1.1.7	N/A	5.0% (28)	16.9% (51)
	B.1.1.58	2.9% (11)	11.8% (66)	1.3% (4)
	B.1.1.70	35.6% (133)	3.9% (22)	1.3% (4)
	B.1.146	2.4% (9)	5.4% (30)	4.3% (13)
	B.1.160	32.6% (122)	2.0% (11)	2.3% (7)
	B.1.177	N/A	N/A	0.3% (1)
	B.1.177.28	N/A	N/A	1.0% (3)
	B.1.177.44	N/A	N/A	0.3% (1)
	B.1.258	2.9% (11)	6.1% (34)	4.6% (14)
	B.1.258.17	18.2% (68)	65.2% (364)	63.6% (192)
	B.1.149	N/A	N/A	0.7% (2)
	B.1.531	N/A	N/A	0.3% (1)
	B.1.533	N/A	0.2% (1)	0.3% (1)
	B.1.565	N/A	N/A	0.3% (1)
	C.35	1.6% (6)	N/A	N/A

a This number is an approximation, as data about the exact work site was not accessible for all of the staff.

### 3.2 Assessment of SARS-CoV-2 introduction events in LTCFs in October 2020

In October 2020, 21 LTCFs with COVID-19 outbreaks were identified in which there were at least two residents with confirmed SARS-CoV-2 infection in a respective LTCF within 14 days. This included 14/21 (66.6%) LTCFs for which Pangolin SARS-CoV-2 lineage designation revealed that the outbreak was caused by one SARS-CoV-2 lineage and 7/21 (33.3%) LTCFs for which multiple variants of SARS-CoV-2 were detected during the outbreak. The co-occurrence of two different lineages were detected in all of these cases: B.1.1.70 and B.1.1, B.1.1.70 and B.1.160, B.1.258.17 and B.1, B.1.258.17 and B.1.258, B.1.1.70 and C.35, B.1.258 and B.1.160, and B.1.258.17 and B.1.1.70.

### 3.3 Complete genome phylogenetic analysis of the first outbreak in one specific LTCF

**Figure 2:**
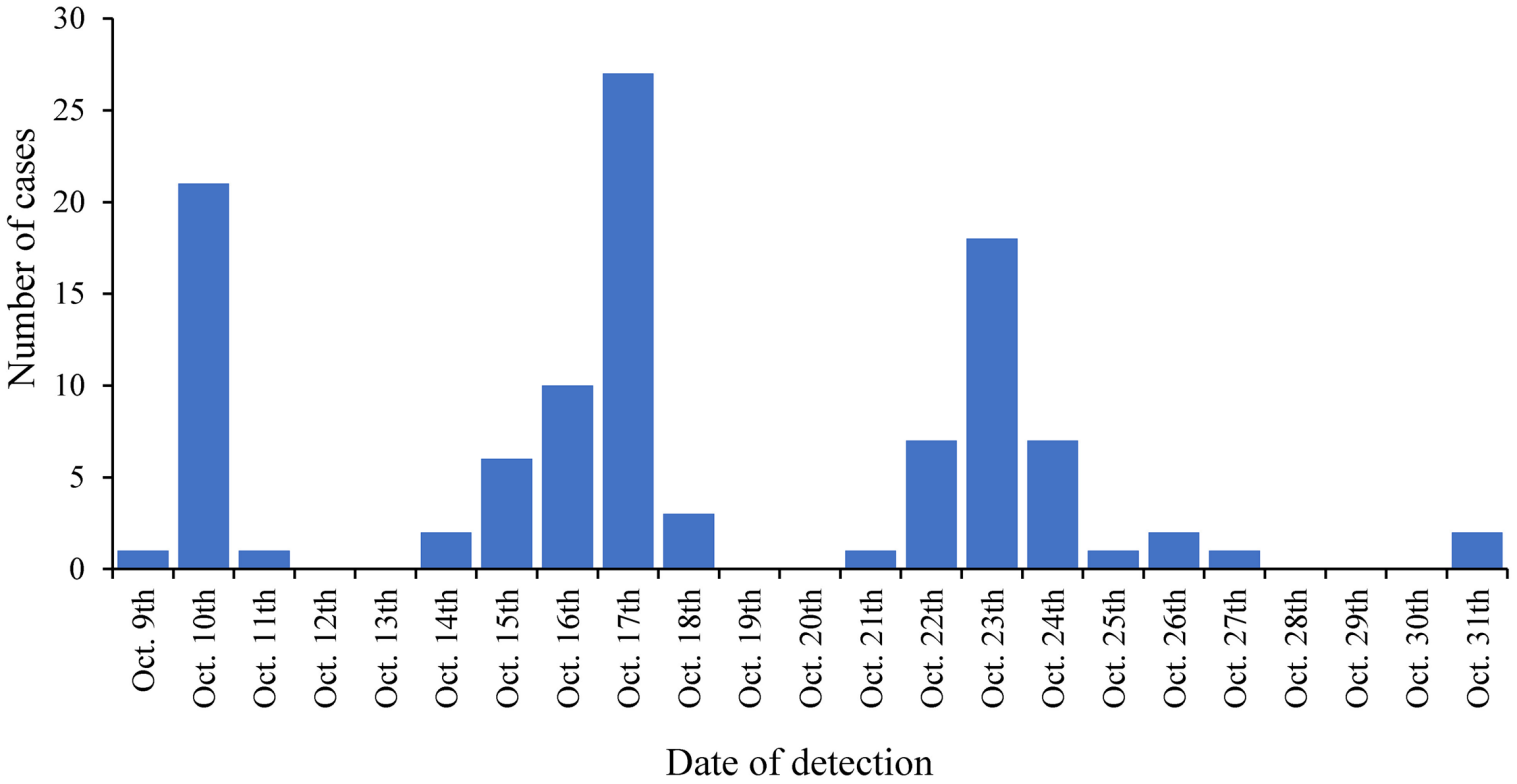
Number of sequenced SARS-CoV-2 cases in a specific LTCF per day in October 2020.

The LTCF with the largest outbreak in October 2020 was selected for an in-depth analysis of SARS-CoV-2 complete genome sequences (genomic data are available at GISAID: EPI_SET_231114ud, https://10.55876/gis8.231114ud, accessed on November 14th, 2023). This LTCF was selected because no SARS-CoV-2 cases were detected prior to this period in this facility (an immunologically naive population) and residents were not vaccinated because the vaccine was not yet available. During this period, 145 cases were confirmed among residents, the first on October 9th, 2020, and then throughout the month with peaks on October 17th, 2020, and October 23rd, 2020, as shown in Figure 2. Among these 75.9% (110/145) were successfully sequenced.

**Figure 3:**
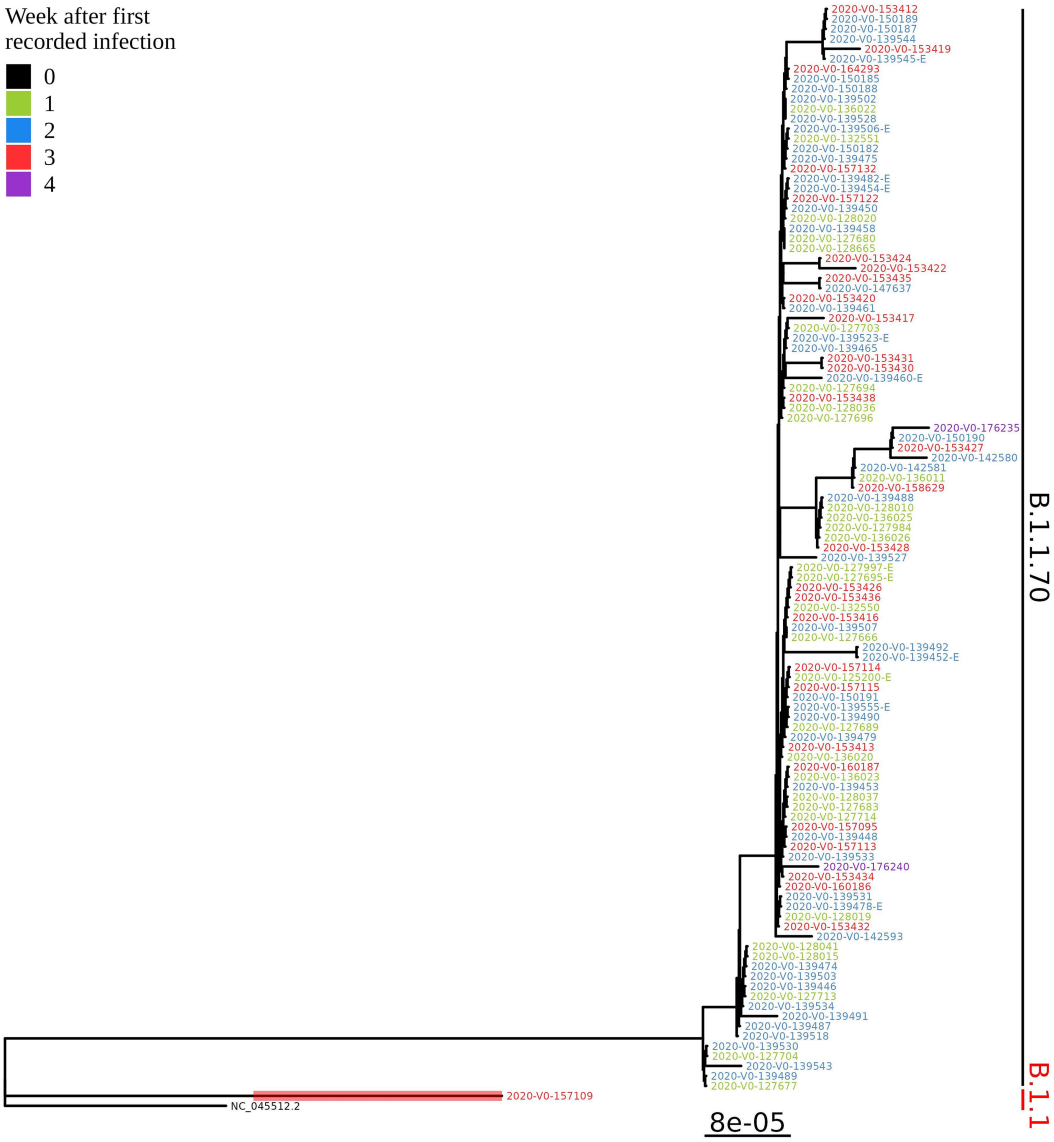
Phylogenetic tree of all SARS-CoV-2 positive residents with successfully obtained complete viral genome sequence within a month (110 samples) from the same LTCF in October 2020. Colours represent the week of detection.

**Figure 4:**
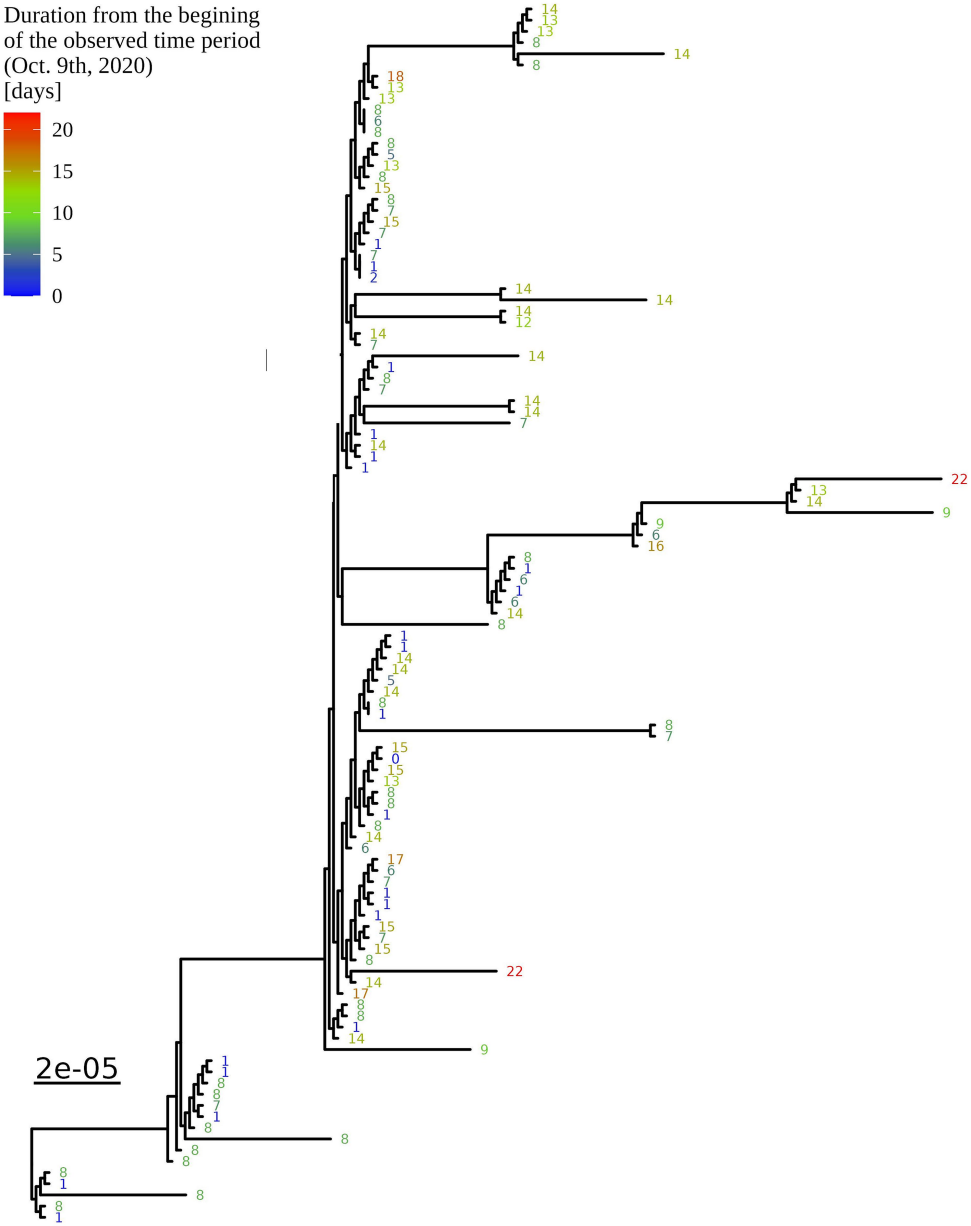
Enlargement of the B.1.1.70 part of the complete SARS-CoV-2 genome analysis phylogenetic tree from one LTCF. The numbers represent delay times in days when the PCR test was positive after the first detected case on October 9th, 2020 (0).

Based on the determination of the SARS-CoV-2 lineage by Pangolin, 99.1% (109/110) of the cases collected during the month belonged to lineage B.1.1.70. The only sample from a different lineage (B.1.1) was collected on October 24th, 2020, clearly indicating an independent introduction event already at the Pangolin level (Figure 3). A detailed analysis of the B.1.1.70 cluster, based on complete genome sequences, indicated that there must have been several introductions of this lineage. The first case detected on day 0 already contains more mutations than samples detected at later time points. Moreover, the samples discovered on the same day are scattered across the phylogenetic tree and contain a larger number of mutations than would be possible considering the SARS-CoV-2 mutation fixation rate (28). On the other hand, some pairs are observed that appear to confirm internal transmission due to an appropriate delay in detection (incubation) and do not show any differences in the genome. In addition, some samples collected around the same day suggest a common source of infection because they are genetically identical (Figure 4).

### 3.4 In-depth mutation profile analysis of the B.1.1.70 subgroup from the selected LTCF

**Figure 5:**
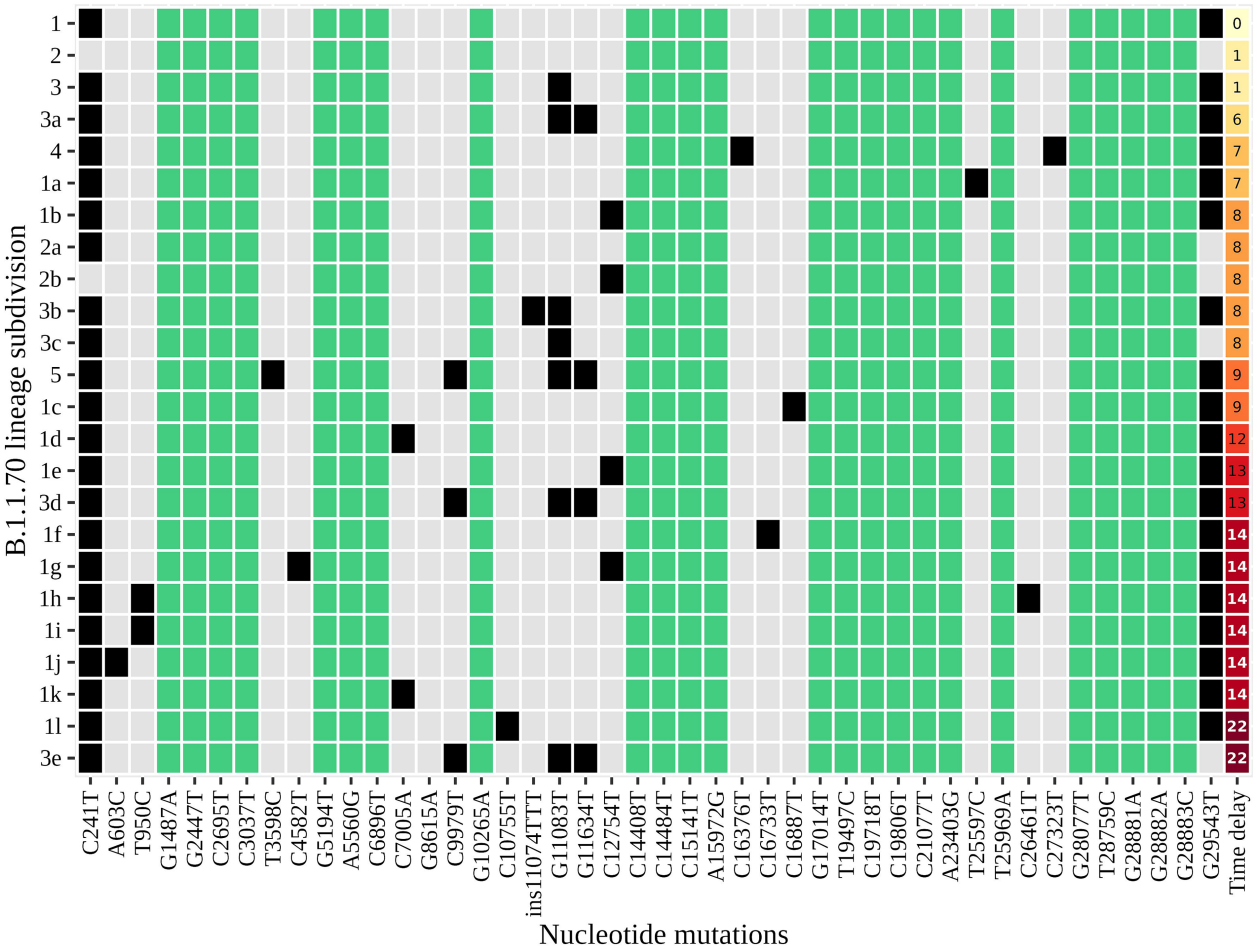
The distinct mutation profile of the B.1.1.70 subgroup in one specific LTCF. The y-axis represents the classification beyond Pangolin, with a sequential number denoting a potential independent introduction event and a letter if the mutation profile represents an expected change within a certain timeframe (possible intra-LTCF transmission). Green represents substitutions that are fixed, grey represents substitutions that differ between samples, and black represents the presence of substitutions in a mutation profile. The last column represents the time delay after the first detection of SARS-CoV-2.

**Figure 6:**
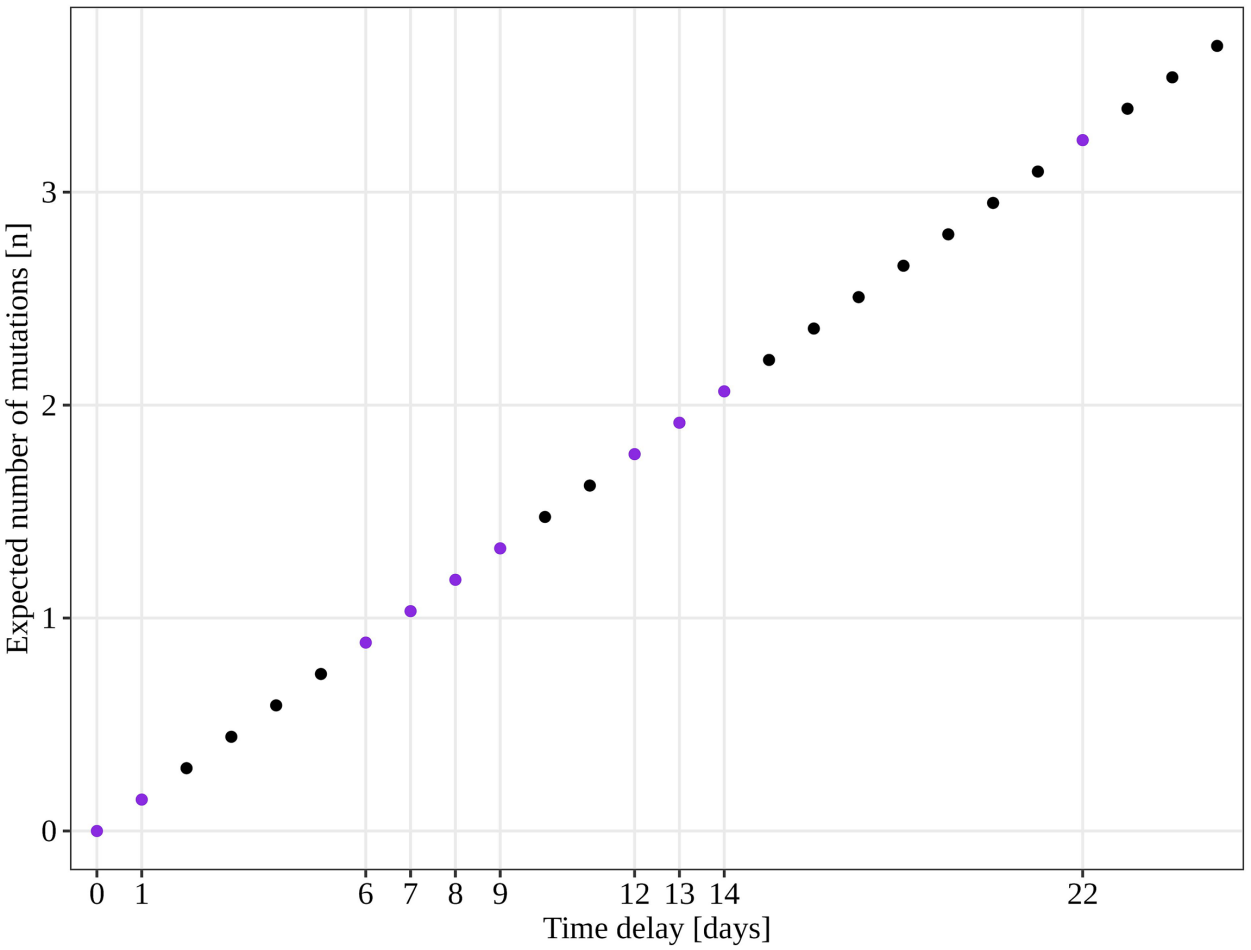
Estimated number of SARS-CoV-2 mutations accumulated in a closed system based on 1.8 × 10^−3^ mutations acquisition per base per year (calculated for the SARS-CoV-2 reference genome NC_045512.2). The purple dots represent the time points observed in our study and the expected number of mutations if only intra LTCF transmission were to occur.

An additional investigation of the mutation profile of the samples of the B.1.1.70 subgroup revealed 24 distinct mutational constellations, suggesting at least five independent introduction events (Figure 5). The distinction between the suspected independent introductions was based on the estimated mutation rate of 1.8 × 10^−3^ substitutions per base per year, which corresponds to one additional mutation per week (28). There are four sample subgroups (designated by the numbers 1–4) with more than one additional substitution already present in the 1st week of investigation (time delay <7 days), indicating independent introduction events. Another possible introduction event is evident on the 9th day with four additional substitutions (mutation profile 5). All other mutation profiles (designated by number and letter; 1a–1 h, 3a–3e) suggest either intra-LTCF transmission or independent introduction events based on the number of new mutations and time delays, but without additional epidemiologic data the source cannot be resolved. The thresholds for suspected individual introduction events are shown in Figure 6.

### 3.5 VOC Alpha rate of introduction into LTCFs

**Figure 7:**
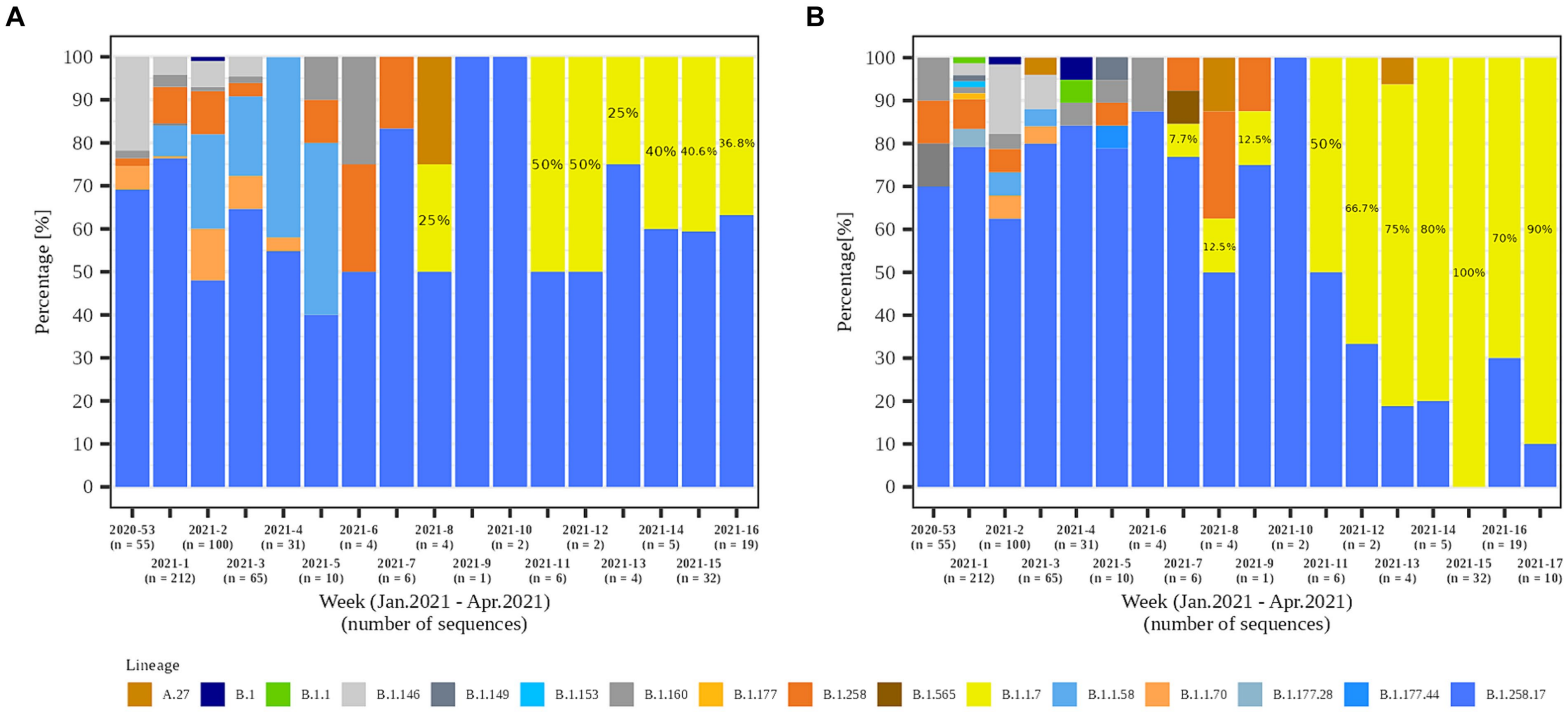
Temporal distribution of detected SARS-CoV-2 lineages in **(A)** LTCF residents and **(B)** LTCF staff per week of detection.

In the second part of the study (January 2021 – April 2021), a total of 79.8% (558/699) and 84.1% (301/358) of the samples from residents and staff from selected LTCFs were successfully sequenced, respectively. At the beginning of the selected period, Alpha was not present in the selected LTCF population (residents and staff) or in the general Slovenian population. The first case of the Alpha variant was detected in the general population on January 7th, 2021. The first Alpha cases in LTCF residents enrolled in the study were detected in week 8/2021. After a 2 week period without detection, Alpha was detected again and accounted for 50.0% of the cases detected. The proportion did not increase until the end of the study period, but it actually decreased slightly, even though the number of positive SARS-CoV-2 tests increased. In contrast, the first Alpha case was detected among LTCFs staff in week 7/2021. After isolated cases in the next 2 weeks and no detection in week 10, Alpha reappeared in week 11. In the following weeks, the prevalence continued to increase until it almost completely replaced the previous lineages. The temporal distribution of detected lineages in LTCF residents and staff from January 2021 to April 2021 is shown in Figure 7.

**Figure 8:**
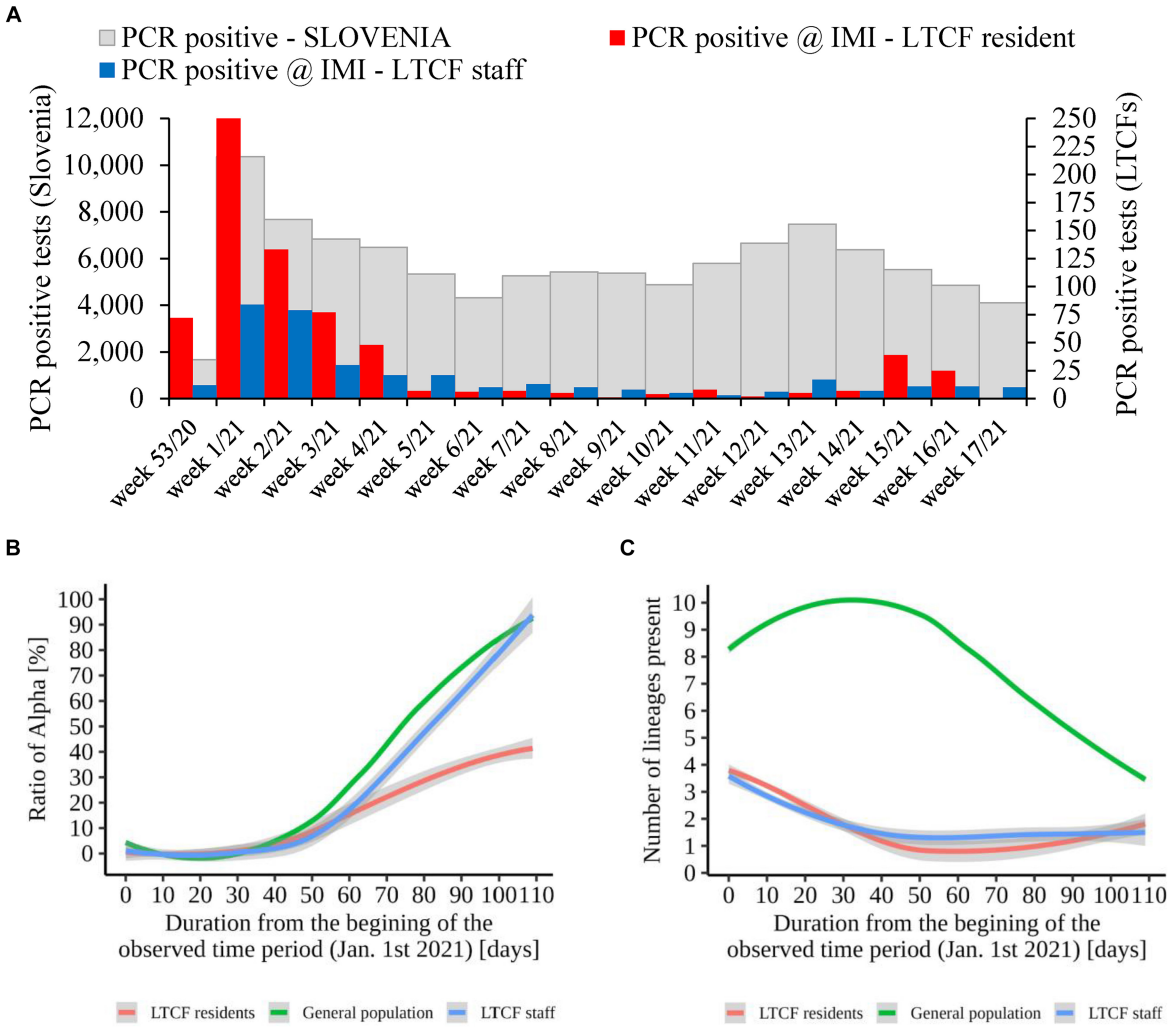
**(A)** Number of SARS-CoV-2 infections in LTCF residents (red) and LTCF staff (blue) and, the total number of PCR positive tests (grey) in Slovenia (all PCR testing sites) per week from January 2021 to April 2021. **(B)** Proportion of VOC Alpha in LTCF residents, LTCF staff, and the general population per day from January 2021 to April 2021. **(C)** Number of different lineages among LTCF residents, LTCF staff, and the general population per day from January 2021 to April 2021. The colored lines represent the median value in the respective populations, and the grey areas represent the 95% confidence interval.

An additional analysis of the proportion of Alpha cases revealed that the growth rate of Alpha cases at the time of introduction to the LTCFs was similar for all three groups observed (i.e., LTCF residents, LTCF staff, and the general population) until the second half of the period analyzed. Thereafter, the growth rate of Alpha was significantly slower for residents than for the general population and LTCF staff. In addition, the analysis of the diversity of lineages (number of different lineages present per day) in the three populations showed that, in the general population, the diversity of circulating lineages increases at the beginning of the study period and starts decreasing toward the middle of the selected period due to Alpha spread. In contrast, lineage diversity decreases in both LTCF residents and staff right at the beginning of the selected period (Figure 8). The number of SARS-CoV-2 infections in LTCFs residents and staff and the number of SARS-CoV-2 infections in the general Slovenian population during the period observed are presented in Figure 8. In some of the weeks observed, the absolute number of cases among LTCF staff and especially among residents was very low.

## 4 Discussion

In this retrospective study, the introduction and subsequent spread of SARS-CoV-2 cases in Slovenian LTCFs were analyzed in detail using NGS, with the aim of providing interdisciplinary data in order to tailor public health interventions during future epidemics. Two time periods based on the epidemiological situation in the country were selected: the initial pandemic phase, when several early lineages were co-circulating in the population, and the period of VOC Alpha appearance in Slovenia, followed by a significant surge in cases.

In the first part of the study, COVID-19 outbreaks in selected Slovenian LTCFs in October 2020 were additionally investigated using complete genome sequencing to identify different variants or introductions of SARS-CoV-2 in each specific outbreak. At that time, Slovenian LTCFs had already been instructed to implement a range of containment strategies such as, the use of personal protective equipment, the establishment of separate zones for residents with confirmed or suspected infections and, monitoring the health status of employees. Because the analyses were conducted at the level of individual LTCFs and exact epidemiological data on all employees in these facilities were not readily available and verifiable (e.g., their contact with residents, their employment in one or more LTCFs, their absence from work, etc.), staff were not included in this part of the study. The findings revealed that the outbreaks in some LTCFs were attributable to two SARS-CoV-2 variants, directly indicating at least two independent sources of infections. However, in the majority of LTCFs, lineage determination at the Pangolin level revealed a single variant, which appears to indicate a single introduction event. However, a more in-depth analysis revealed that instances in which a single Pangolin variant was identified as a cause of an outbreak do not necessarily point to a single SARS-CoV-2 introduction event. In fact, complete genome phylogenetic and mutation profiles analysis of samples from the largest LTCF outbreak in October 2020 showed multiple introductions of the SARS-CoV-2 into the LTCF. Such findings would not have been possible based on the Pangolin lineage designation alone. Moreover, comprehensive contact tracing, which in certain cases could enable the acquisition of such data, is often difficult to carry out in critical situations due to overburdened staff and it tends to be less reliable compared to data acquired faster through sequencing. Obtained data is consistent with some previously published studies that identified several separate introductions of SARS-CoV-2 within one LTCF (29–32). This highlights the importance of not only implementing strategies to limit the spread of infection within LTCFs, but also taking measures to reduce the likelihood of further introduction of the virus from external sources. Although the potential sources of virus introduction into LTCFs are fairly well known (32), based on our results, measures to prevent such instances could include not only the implementation of procedures (33), but also rapid molecular diagnostics coupled with timely available sequencing, as it enables acquiring additional information for future tailoring of public health measures in such environments. Moreover, in the future, sequencing with rapid turnover time could allow for individualized interventions for specific VOCs, if such differentiated actions would have evidence-based background.

The second part of the study aimed to study the substitution of the predominant variants by the emerging, more transmissible VOC Alpha and to ascertain the earliest instance of its introduction in the LTCFs. We also wanted to determine whether a new VOC could spread faster due to the closed environment of LTCFs and the more immunologically vulnerable population. Therefore, the period from January to April 2021 was chosen to cover the time from the discovery of the Alpha variant in Slovenia to the time it became dominant in the general population. This timeframe allowed us to capture the initial cases of the Alpha variant in LTCFs and investigate its subsequent spread. The analysis was not conducted at the level of individual LTCFs, but only by selected population groups as a whole (general population, LTCF residents, and LTCF staff). As expected, the staff cohort exhibited a greater array of different variants than the LTCF residents. This result is not surprising because the former typically have more ongoing interactions outside the facility, exposing them to a wider range of infection opportunities. However, this fact makes employees an important source for the introduction of different variants, which is why they should strictly adhere to all recommended infection prevention and control measures. This is particularly important when a new variant emerges that is more immunologically evasive and can easily re-infect individuals (34). The first case of the Alpha variant in the Slovenian general population was detected approximately a month and a half prior to its detection in the first LTCF resident. At that time, the proportion of the Alpha variant in the general population was close to 15% (35), which may indicate that existing interventions, non-pharmaceutical measures, and vaccination, as well as immunity from prior infections, have slightly delayed the introduction and spread of this VOC in LTCFs.

The assessment of the VOC Alpha proportion showed an initial similarity between the three cohorts observed during the early stages of the spread of this variant, followed by a significant acceleration of the growth trajectory among the general population and LTCF staff in contrast to LTCF residents. At the end of the period observed, when Alpha was already dominant in the general population and LTCF staff, it still barely reached 40% among residents. Slovenia had a very good outcome in the first wave of the pandemic, with several SARS-CoV-2 B lineages circulating. However, in the late summer of 2020, variant B.1.258.17 emerged and spread rapidly throughout the country, causing high mortality (36), partially also because of higher pathogenicity and fitness of the variant. In addition, the vaccination campaign started at the end of December 2020, when LTCF residents and healthcare professionals were the priority groups for vaccination. Exact data on the immunization coverage of Slovenian LTCF residents in January to April 2021 are not available at a national level because uniform reporting to the National Institute of Public Health (NIPH) started in September 2021. However, a questionnaire conducted by the Association of Social Institutions of Slovenia among LTCFs showed that, by mid-June 2021, the average vaccination coverage among Slovenian LTCF residents with at least one dose was approximately 83%. Based on the later assessment of vaccination coverage by the NIPH, in which the vaccination coverage was broadly comparable between LTCFs, we assume that this was also the case with these questionnaire-based results. In contrast, by mid-June 2021 the proportion of the general population over 18 years old vaccinated with at least one dose and the proportion of fully vaccinated individuals was 46 and 34%, respectively (37). The collective contribution of the high prevalence of prior infections with B.1.258.17 and vaccination efforts among residents together with several implemented mitigation strategies (cohorting residents with confirmed or suspected infections, use of personal protective equipment, control measures that allow safe visits, monitoring of health status of staff and residents, hygiene precautions, cleaning and disinfection, etc.) hindered the introduction and spread of the Alpha variant in LTCFs. The decrease in the number of lineages present in both LTCF residents and staff in the early part of the observation period and the simultaneous increase in the general population may also indicate that this closed environment was at least reasonably well protected from additional introduction of different variants, probably by the same factors as mentioned above.

The findings in both the first and second parts of the study emphasize the importance of effective pharmaceutical and non-pharmaceutical preventive measures to curb the introduction of SARS CoV-2 into LTCFs while controlling the spread of infection within them. This is particularly important because residents of LTCFs are at high risk of severe disease progression and increased mortality due to their underlying health conditions, frailty, or advanced age, in addition to the confined living spaces inherent to these facilities (38, 39). Nonetheless, the implementation of non-pharmaceutical interventions in LTCFs requires caution and careful consideration because certain interventions, although intended to protect life, can significantly affect the overall quality of life of residents (40, 41). For example, if implemented non-pharmaceutical interventions restrict physical, face-to-face interactions or affect the availability of healthcare services, this may exacerbate the social vulnerability of LTCF residents (33). Thus, should a novel SARS-CoV-2 variant or any other potentially threatening pathogen emerge for which the impact and efficacy of vaccination remain uncertain, LTCF residents should be prioritized for both pharmaceutical and non-pharmaceutical interventions, among which interventions that exert the least negative implications on their quality of life should be prioritized.

The limitations of the study should be considered when interpreting these results. The first part of the study included all LTCF residents whose infection was rtRT-PCR confirmed in October 2020 and who were part of outbreaks, as defined in the Materials and Methods section. However, because certain outbreaks persisted over extended periods of time and spanned months in which a dominant variant was already present, individuals whose infections were confirmed after October 2020 were not included. Therefore, the actual number of different variants and introductions that contributed to a LTCF outbreak could be higher if the entire pool of additional residents and a longer study period had been included. Nevertheless, we believe that the approach employed fulfilled its intended purpose to identify the potential for multiple introductions within a single outbreak. An important limitation of the second part of this study is that the number of confirmed cases among LTCF staff and especially residents included in the study was very low in some of the weeks observed, thus broadening the confidence interval of observed data. However, these numbers still provide an accurate description of the COVID-19 epidemiological situation in LTCFs, as they include all rtRT-PCR confirmed residents and staff of LTCFs, with no additional exclusion criteria. Although the IMI has performed on average nearly half of all SARS CoV-2 PCR tests in the country at any given time during the pandemic it mainly covers the central, western, and southern geographical and statistical regions of Slovenia. Because the LTCF staff included in the second part of the study were defined as all persons that had a positive SARS CoV-2 test in the selected period and were registered in the national digital administrative database as employees in LTCF, these criteria also include all those that were not necessarily involved in the outbreaks or had any contact with residents because these data were not readily available. However, the decision to include staff in this segment, despite the aforementioned limitations, was justified by the fact that, due to the intense workload during this period, most of these LTCFs staff infections would have had some form of impact on the workplace.

Despite the aforementioned limitations, we believe that the data obtained, combined with the additional body of knowledge from the LTCFs, could prove useful in future efforts to control communicable diseases. We have shown that sequencing is a powerful supplementary tool to distinguish between purely internal spread and the occurrence of additional external introductions in the context of outbreaks in closed and high-density environments. In this context, our research supports the use of complete genome sequencing as a valuable tool because these types of data combined with epidemiological data can provide useful information for future public health implications not only limited to COVID-19, but also for other potential threats that may emerge in the future.

As revealed by this study, COVID-19 outbreaks in Slovenian LTCFs have been caused by intra-facility transmission as well as several different SARS-CoV-2 introductions. Substitution of the current variants with VOC Alpha appeared to be slower in LTCF residents than in LTCF staff or the general population, possibly reflecting the effectiveness of the non-pharmaceutical interventions implemented in combination with the uptake of the vaccine among residents, as well as at least partial immunity due to prior infections and vaccination efforts. Given the substantial impact of COVID-19 on LTCFs, it is imperative to prioritize measures in such facilities and adopt timely and proportionate mitigation strategies to effectively protect the residents from SARS-CoV-2, including new VOC variants and any other potential threats that may emerge in the future. We show that the data obtained by genome sequencing, in conjunction with epidemiological data, can help identify the need for future improvement of additional control measures aimed at protecting LTCF residents from other impeding threats, extending beyond COVID-19.

## Data availability statement

The datasets presented in this study can be found in online repositories. The names of the repository/repositories and accession number(s) can be found at: https://gisaid.org/, EPI_SET_231114ud.

## Ethics statement

The studies involving humans were approved by National Medical Ethics Committee, Ministry of Health, Republic of Slovenia (0120 211/2020/7). The studies were conducted in accordance with the local legislation and institutional requirements. Written informed consent for participation was not required from the participants or the participants' legal guardians/next of kin because the manuscript contains no identifying information or images and the results have no direct impact on patient's management or health.

## Author contributions

RK: Data curation, Formal analysis, Investigation, Validation, Visualization, Writing – original draft, Writing – review & editing. MG: Data curation, Formal analysis, Investigation, Methodology, Validation, Writing – original draft, Writing – review & editing. AS: Data curation, Formal analysis, Investigation, Methodology, Visualization, Writing – original draft, Writing – review & editing, Software. SZ: Formal analysis, Methodology, Writing – review & editing, Software. DV: Methodology, Writing – review & editing. KKK: Methodology, Writing – review & editing. MF: Conceptualization, Methodology, Resources, Supervision, Writing – original draft, Writing – review & editing, Project administration. MP: Conceptualization, Funding acquisition, Resources, Supervision, Writing – review & editing, Project administration. TA-Ž: Conceptualization, Funding acquisition, Resources, Supervision, Writing – review & editing, Project administration. MK: Conceptualization, Funding acquisition, Investigation, Resources, Supervision, Validation, Writing – review & editing, Project administration.
